# Complete Response to Nivolumab‐Relatlimab Following Progression of Invasive Sinonasal Mucosal Melanoma on First‐Line Nivolumab‐Ipilimumab: A Case Report

**DOI:** 10.1002/cnr2.70544

**Published:** 2026-04-10

**Authors:** Reema Patel, Louisa Post‐Zwicker, Varinder Kaur

**Affiliations:** ^1^ University of Pittsburgh Medical Center Pittsburgh Pennsylvania USA; ^2^ University of Virginia Cancer Center Charlottesville Virginia USA

**Keywords:** immunotherapy, mucosal melanoma, relatlimab

## Abstract

**Background:**

Mucosal melanoma (MM) is a rare and aggressive subtype of melanoma with a notably worse prognosis than cutaneous melanoma (CM) due to its occult presentation and unique molecular profile. For advanced cases, immunotherapy has improved outcomes in recent years, although more significantly for CM. The recently approved combination of nivolumab and relatlimab has emerged as a promising option for advanced melanoma and a potential alternative to the well‐established nivolumab‐ipilimumab regimen. Due to the rarity of MM often limiting thorough evaluation in trials, there remains a lack of guidance regarding the most appropriate regimen for optimal patient outcomes.

**Case:**

A 70‐year‐old male with T4aN0M0 sinonasal MM was initiated on neoadjuvant nivolumab‐ipilimumab therapy; however, disease progression soon after rendered the tumor unresectable. Despite undergoing 4 cycles, metastatic progression ensued, necessitating a shift to nivolumab‐relatlimab therapy. Following 8 cycles of nivolumab‐relatlimab alongside radiation to metastatic sites, the patient achieved a complete response, with no signs of active disease. Unfortunately, during a seven‐week treatment interruption due to suspected immune‐related gastrointestinal toxicity requiring corticosteroids, routine PET‐CT monitoring revealed findings suspicious for new metastasis. Despite one additional cycle of immunotherapy, the patient's functional status deteriorated. Although repeat imaging showed stable disease, the patient elected to transition to supportive care without further treatment.

**Conclusion:**

This case exemplifies the use of nivolumab‐relatlimab in achieving a complete response in a patient with advanced sinonasal MM that progressed after nivolumab‐ipilimumab. While further investigation is needed to clarify the comparative efficacy of existing combination immunotherapy options, this report highlights nivolumab‐relatlimab as a promising option with a potentially favorable safety profile in this rare and aggressive melanoma subtype.

## 1 Introduction

Mucosal melanoma (MM) is a rare and particularly aggressive subtype, constituting less than 2% of all melanoma diagnoses. It can originate from a variety of sites, including the head and neck regions, upper and lower gastrointestinal tracts, and urogenital tracts. MM is known for its occult presentation, which complicates early detection, often leading to diagnosis at more advanced stages compared to other subtypes [1, 2]. MM also exhibits a distinct molecular profile, with a lower frequency of BRAF mutations, reduced PD‐L1 expression, and a variable rate of KIT mutations depending on anatomical site [3, 4, 5]. These molecular differences contribute to MM's limited responsiveness to targeted therapies and immunotherapy compared to cutaneous melanoma (CM). Clinically, MM patients experience poorer outcomes with a 5‐year overall survival (OS) of approximately 25% [6, 7] compared to > 90% for CM [8].

Historically, treatment for localized MM has relied on surgical excision with the goal of achieving clear margins. Unfortunately, despite surgery, many patients still develop local recurrence or metastases. Additionally, due to the anatomic complexity of certain sites and their proximity to critical structures, radical surgical management is not always be feasible, necessitating the use of radiotherapy or systemic therapy. For unresectable or metastatic disease, the emergence and growing utilization of immunotherapies (i.e., CTLA‐4 and PD‐1 inhibitors) has yielded substantial improvements in survival outcomes, albeit with greater effect in CM [1, 2, 9]. A sub‐analysis of the CheckMate 067 trial demonstrated that patients with MM had higher objective response rates (ORR) and OS when treated with nivolumab and ipilimumab together compared to monotherapy [10]. Notably, a pooled analysis by D'Angelo et al. reported a lower objective response rate (ORR: 37% vs. 60%) and shorter progression‐free survival (PFS: 5.9 vs. 11.7 months) in patients with MM versus CM, respectively. More recently, in 2022, the US Food and Drug Administration (FDA) approved the use of fixed‐dose relatlimab, a checkpoint inhibitor targeting LAG‐3, in combination with nivolumab for patients with unresectable or metastatic melanoma following results from the Phase II/III RELATIVITY‐047 trial, which showed superior PFS with the combined regimen over nivolumab alone.

Currently, there are no head‐to‐head trials directly comparing the efficacy of these two combination immune checkpoint inhibitor (ICI) regimens for advanced melanoma. Additionally, in the absence of standardized guidelines, treatment decisions often vary by institution or provider preference. There is a critical need to improve the prognosis for MM, yet its rarity has hindered comprehensive evaluation of both existing and investigational therapies. While clinical trials remain ongoing, retrospective analyses and patient cases may provide valuable insights into selecting the most appropriate treatment regimen thereby optimizing outcomes for patients. To contribute to the understanding of regimen selection, we present the case of a patient diagnosed with locally advanced, unresectable T4aN0M0 sinonasal MM who achieved a complete response on nivolumab‐relatlimab therapy after initial progression on first‐line nivolumab‐ipilimumab.

## 2 Case

**FIGURE 1:**
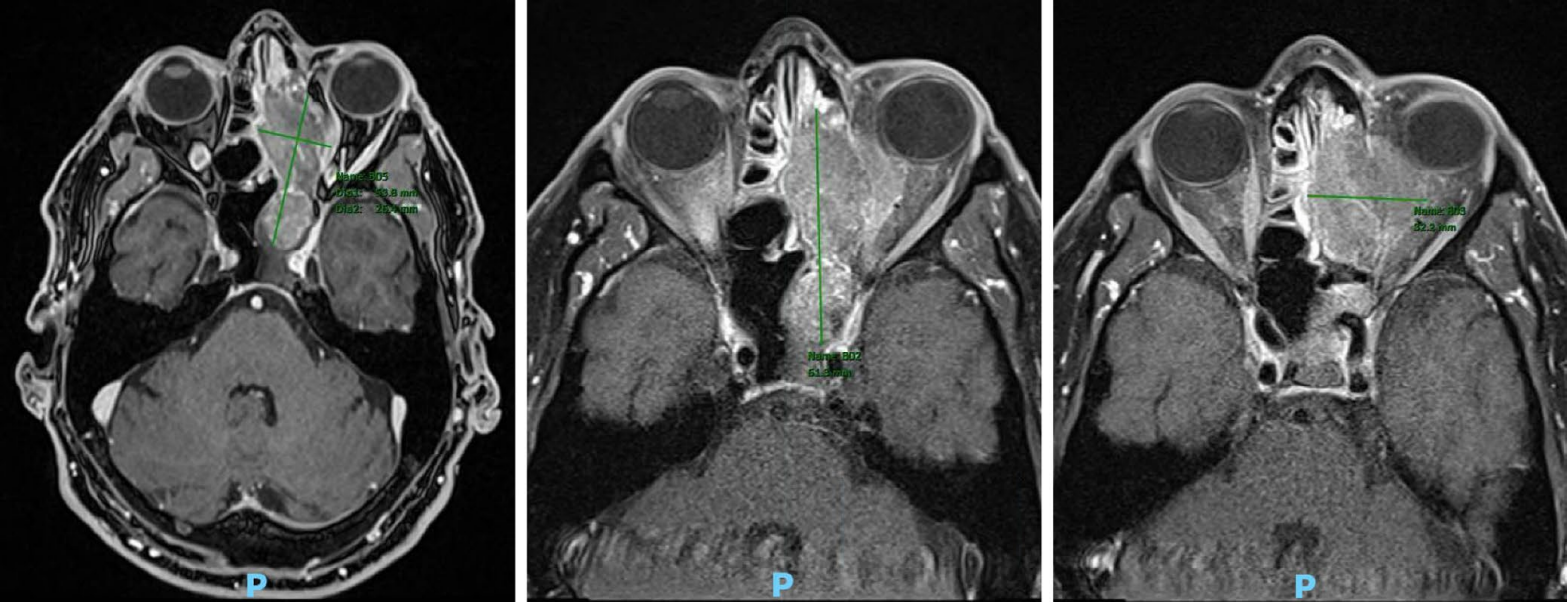
MRI orbit face neck imaging showing a large locally advanced left‐sided sinonasal mass involving the left ethmoid, left sphenoid, and left superior nasal cavity with gross invasion of the left medial orbit where there is a left optic nerve compression. There is also evidence of invasion of the anterior skull base with suspicious early dural invasion. The sphenoid component of the tumor is bordering the left precavernous/cavernous internal carotid artery and left optic canal without invasion.

A 70‐year‐old man with a history of hereditary hemochromatosis, excised melanoma, and basal cell carcinoma presented to the University of Virginia emergency department in 2022 for worsening headache, blurry vision, ear pain, sinus congestion, and an isolated episode of vertigo. Imaging studies revealed a large left‐sided sinonasal mass involving the ethmoid, sphenoid, and superior nasal cavities with invasion into the orbits and skull base, as well as optic nerve compression (Figure 1). Biopsy was performed, and final pathology confirmed a stage T4a MM. Immunohistochemistry for BRAF (V600E) and PDL‐1 were negative. PET‐CT identified a locally advanced 4.5 cm hypermetabolic sinonasal mass and an indeterminate minimally hypermetabolic left level IIA sub‐centimeter lymph node with no evidence of distant metastasis.

The patient's symptoms progressed quickly with increased eye pain and irrigation, diplopia, and headache, although his ECOG performance status remained at 1. The patient began neoadjuvant immunotherapy with nivolumab (1 mg/kg) and ipilimumab (3 mg/kg). After his first cycle, the eye pain continued to worsen, and repeat magnetic resonance imaging (MRI) showed a significant increase in tumor size with mass effect on the left globe causing proptosis and near‐complete encasement of the optic nerve. Additionally, there was new intracranial extension with dural invasion into the left antero‐inferior frontal lobe, tumor surrounding the cavernous left internal carotid artery, and involvement of the pterygopalatine fossa. There was no evidence of distant intracranial metastasis or cervical lymphadenopathy within the visualized neck. With progression to stage T4b, the tumor was deemed unresectable, and the patient continued immunotherapy with palliative radiation to the left sinonasal melanoma mass at a dose of 50–55 Gy in 20 fractions at 2.5–2.75 Gy per fraction delivered once daily.

The patient completed 3 cycles of nivolumab‐ipilimumab before developing a Grade 2 immune‐related rash that required steroid treatment. The rash improved with 0.5 mg/kg of prednisone; however, it recurred after tapering, requiring an additional extended course for full resolution. Following one additional cycle of nivolumab‐ipilimumab, there were unfortunately new lesions in the right adrenal nodule (1.2 cm) and left acetabulum (1.2 cm) found on repeat PET‐CT imaging concerning for metastatic progression, despite improvement in the primary mass. The decision was made to switch to nivolumab‐relatlimab, 480 mg every 4 weeks, with concurrent radiation therapy of the right adrenal gland metastasis to a dose of 35–40 Gy in five fractions and the left acetabular metastasis to a dose of 30 Gy in five fractions.

**FIGURE 2:**
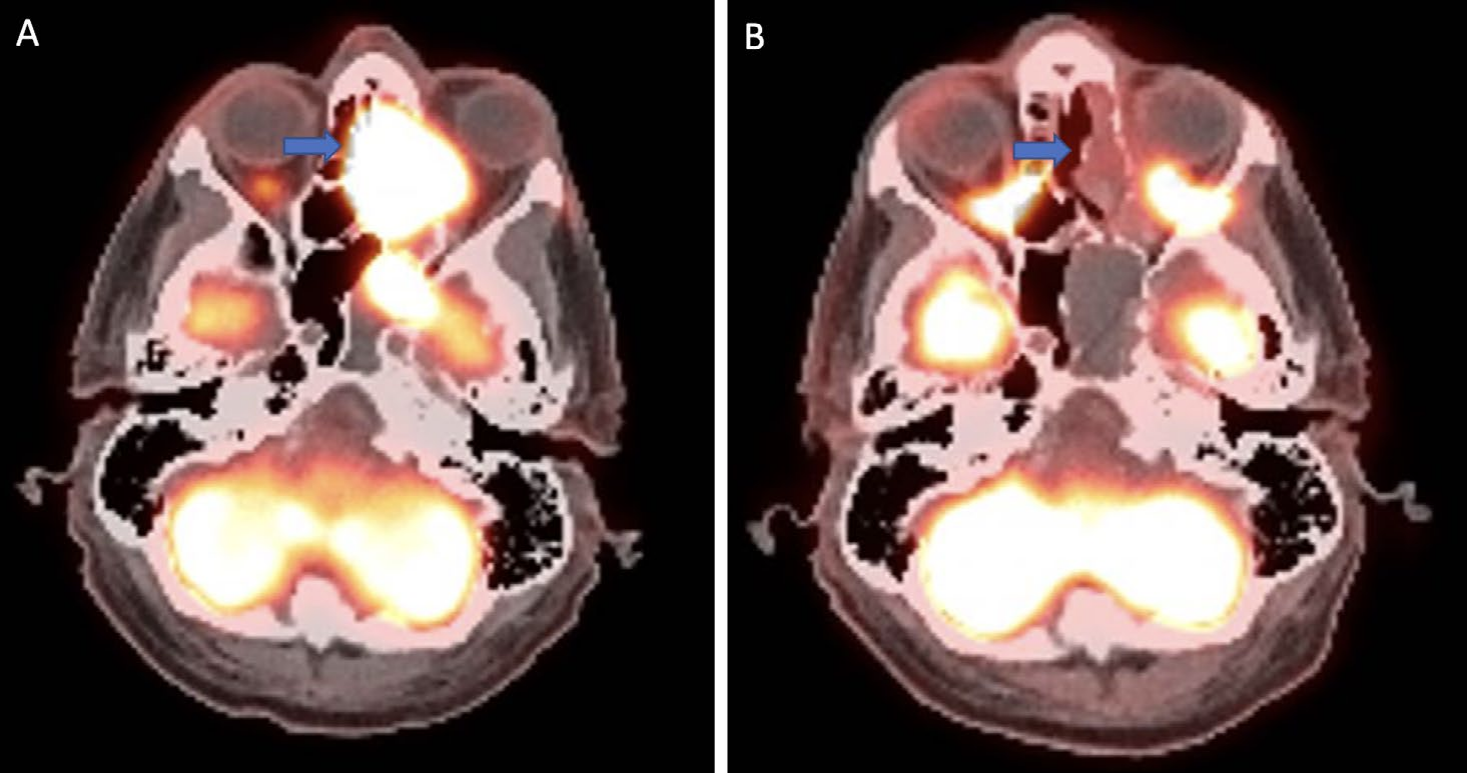
(A) Baseline FDG‐PET imaging demonstrating large hypermetabolic lesion in left nasal cavity. (B) FDG‐PET showing no FDG‐avid lesions in the nasal cavity after cycle 7.

The first few cycles were tolerated relatively well; however, his treatment course was notable for the development of pulmonary emboli requiring apixaban but no interruption of nivolumab‐relatlimab. Interval PET‐CT 3 months after treatment initiation showed improvement to his metastatic lesions; however, with a new left level IIB cervical lymph node measuring 1.6 × 1.1 cm along with a more conspicuous right level IB sub‐centimeter lymph node. Three months later, PET‐CT revealed a new 1.1 cm hypermetabolic aortopulmonary (AP) window lymph node and slightly increased size and metabolic activity of right level IB lymphadenopathy. Fortunately, PET‐CT at 9 months was without signs of active disease in the nasal cavity (Figure 2), exhibiting additional resolution of previous hypermetabolic left cervical lymphadenopathy and a decrease in size and metabolic activity of left AP window nodes.

**FIGURE 3:**
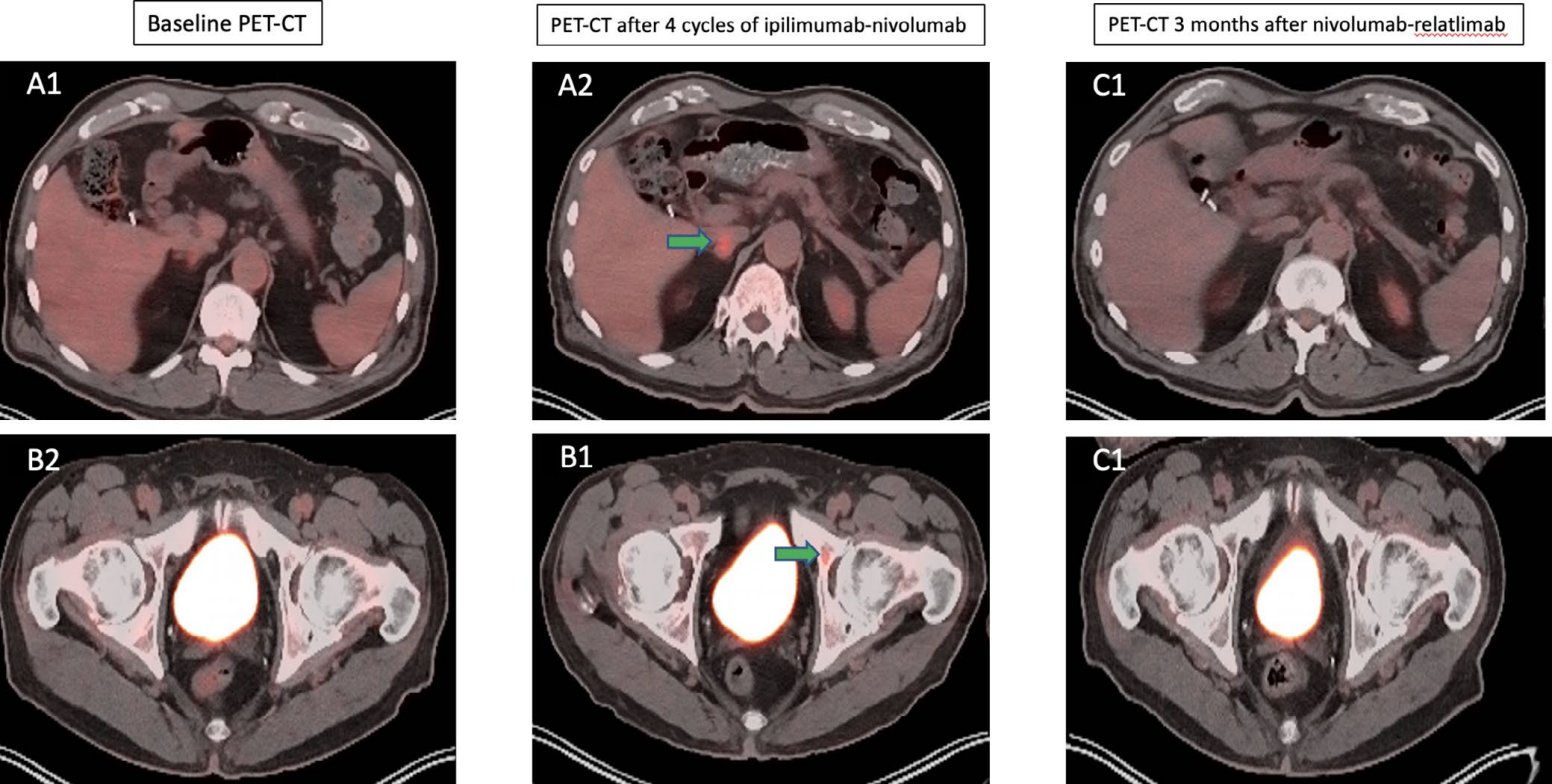
PET‐CT images demonstrating disease status before and after ipilimumab‐nivolumab and nivolumab‐relatlimab regimens. (A2 and B2) FDG‐PET showing new hypermetabolic right adrenal (A2) and left acetabular lesions (B2) in comparison to baseline images (A1 and B1) showing no such lesions, thus consistent with distant disease progression after 4 cycles of ipilimumab plus nivolumab. (C1 and C2) Interval resolution of right adrenal and left acetabulum hypermetabolic activity 3 months following nivolumab plus relatlimab therapy.

The patient continued to have radiographic response with monthly courses of nivolumab‐relatlimab (Figure 3). Prior to the ninth cycle, the patient presented with nausea, vomiting, and orthostatic dizziness for 1 week, preventing him from tolerating anything by mouth. There was a concern for immunotherapy‐related gastrointestinal toxicity; therefore, treatment was held, and the patient was admitted to the hospital. While admitted, workup revealed a mild leukocytosis, normal lipase, normal transaminases, and CT abdomen/pelvis showing uncomplicated diverticulitis. The patient was started on 1 mg/kg prednisone daily, along with famotidine and trimethoprim‐sulfamethoxazole. He also received vancomycin due to discordant *C. difficile* testing. His symptoms improved over the course of 1 week, and prednisone was subsequently tapered.

During the steroid taper, the patient underwent repeat PET‐CT which unfortunately showed a new hypermetabolic right lower paratracheal lymph node, suspicious for metastasis, and a sub‐centimeter lymph node showing new mild hypermetabolic activity. After treatment interruption for 7 weeks, Cycle 9 was administered. Over several weeks, the patient developed worsening fatigue, emesis, dyspnea, and presyncope leading to multiple falls and admission for failure to thrive. Workup was unremarkable, and clinical management involved providing supportive care and holding further immunotherapy. While repeat CT imaging demonstrated stable disease, given symptom progression and declining functional status, the patient elected to proceed with optimal supportive care.

## 3 Discussion

To the authors' knowledge, this is the first report describing complete response with second‐line nivolumab‐relatlimab therapy for advanced sinonasal MM. The patient initially received nivolumab‐ipilimumab, intending to undergo surgical resection of the 4.5 cm tumor; however, she experienced metastatic progression after 4 cycles, prompting the decision to switch therapy. The primary tumor and its metastatic sites responded well, with no signs of active disease after 8 cycles of nivolumab‐relatlimab. Despite this promising outcome, the suspected development of immune‐related colitis led to a 7‐week treatment interruption and corticosteroid therapy, after which signs of disease progression were found on interval imaging. Another cycle of nivolumab‐relatlimab was administered, but unfortunately, the patient's functional status gradually declined, leading to transition to solely supportive care.

Since the approval of nivolumab‐relatlimab in 2022, there are very few studies reporting outcomes of this regimen in the specific context of MM and none following progression after first line nivolumab‐ipilimumab therapy [11, 12]. For example, one case described near clinical complete response of locally advanced conjunctival melanoma after starting nivolumab‐relatlimab as first‐line therapy [12]. Another case illustrated complete abscopal response in a locally advanced sinonasal MM refractory to first‐line nivolumab monotherapy after the concurrent use of stereotactic body radiation therapy and nivolumab‐relatlimab [11]. Other studies evaluating the nivolumab‐relatlimab regimen for advanced melanoma, including a mono‐ versus combination therapy lead‐in approach [13], are ongoing; although, whether specific subgroup analyses for MM can reasonably be performed is unclear. Overall, there is a clear paucity of data on the direct study of nivolumab‐relatlimab combination therapy in MM—in part due to the rarity of this melanoma subtype—therefore much of our current understanding of its clinic efficacy in MM has been obtained from case reports and indirect comparisons.

While the superiority of combination immunotherapy over monotherapy for MM has been established in clinical trials [14, 15], there is a lack of evidence comparing the existing options for combination therapies head‐to‐head. Of note, in the recent Relativity‐047 trial, ORR could not be formally tested; therefore, nivolumab‐ipilimumab, per results of the CheckMate 067 trial, tends to be chosen as first‐line treatment more frequently. Interestingly, confirmed ORR per blinded independent central review showed an ORR of 43.1% for nivolumab‐relatlimab versus 32.6% for nivolumab alone despite no insight on significance. One network meta‐analysis summarizing outcomes in 9070 metastatic melanoma patients from 18 randomized trials—albeit with no subgroup analysis of non‐CM due to the rarity of reported data—demonstrated a similar PFS and ORR with nivolumab plus relatlimab compared to nivolumab plus ipilimumab, with the former appearing to have a better safety profile [16]. On the other hand, a recent indirect comparison of findings from the Relativity‐047 and CheckMate 067 trials demonstrated that while nivolumab‐relatlimab showed similar efficacy to nivolumab‐ipilimumab in the overall population, subgroup analysis appeared to favor the latter in MM, though the authors preface that their results should be interpreted with caution given the small sample size [17]. Overall, there is still a need for additional investigation to clarify the comparative efficacy of these two treatment options.

Beyond their distinct immunoregulatory targets, the downstream anti‐tumor response of LAG‐3 and CTLA‐4 inhibition in MM may differ based on characteristics of the tumor microenvironment itself. Studies have shown an association between mutational load and clinical efficacy with CTLA‐4 blockade, but not PD‐1 or LAG‐3 blockade [3, 18, 19]. MM has a lower mutational burden and reduced neoantigen load compared to CM, which may therefore result in reduced efficacy of anti‐CTLA‐4 agents.

It is important to also note the potential synergy of ICI therapy and radiotherapy (RT), as the described patient received palliative radiation to both primary and metastatic sites in addition to immunotherapy. Several studies have established the synergist effects of combining ICI and RT; in the context of MM, current data primarily supports the enhancement of local control, as observations describing abscopal effects remain limited. For example, Kim et al. reported a 1‐year target lesion (primary and/or metastatic) control rate of 94.1% with anti‐PD1 + RT treatment compared to 57.1% and. 25% in RT‐ and ICI‐alone groups, respectively, with only one patient in the combined group achieving CR [20]. The patient described in this case report experienced a profound and sustained response at the primary site with ICI + RT supporting the observations seen in prior studies [21].

With immunotherapy use, there is an established risk for immune‐related adverse events. The patient described in this report experienced significant gastrointestinal side effects thought to be associated with ICI use and thus required steroid intervention, although he was also treated to rule out an infectious cause. While combination therapies are associated with a higher risk of toxicity compared to monotherapies, nivolumab‐relatlimab may be a safer option to reduce the risk of toxicity based on trial data comparing the safety profiles of dual ICI regimens in melanoma [16].

One potential limitation of this case report was the lack of durable treatment response seen in the reported patient, as disease progression occurred after 8 cycles. This observation is confounded by the 7‐week treatment interruption the patient experienced for treatment of suspected ICI‐colitis. Interestingly, surveillance imaging prior to the immune‐related adverse event was without evidence of progression. It is unclear if the duration of treatment interruption was clinically significant enough to contribute to the observed relapse of disease. Observational data, while limited, has suggested that MM itself is a strong, statistically significant risk factor for disease recurrence post‐ICI discontinuation after CR compared to other melanoma subtypes, with a shorter time to relapse as well [22]. Whether the reported patient's cancer progressed due to treatment interruption, the inherent aggressive nature of MM, eventual acquired resistance to treatment, or a combination of these factors is difficult to ascertain. Regardless, it remains the case that nivolumab‐relatlimab therapy yielded some duration of benefit for the patient, as evidenced by CR at 9 months, after non‐response to first‐line therapy.

## 4 Conclusions

This report highlights the promising utility of nivolumab‐relatlimab for advanced MM as evidenced by the complete response experienced by a patient who developed metastatic disease that was refractory to an initial regimen of nivolumab‐ipilimumab. This finding is noteworthy given limited investigational data of combined regimens in this rare melanoma subtype that is complicated by historically poor response rates. While the initiation of nivolumab‐relatlimab as first‐line therapy over nivolumab‐ipilimumab remains under debate, it will be important to further investigate this question in prospective trials, with particular attention to whether anti‐LAG‐3 therapy exhibits superior efficacy over anti‐CTLA‐4 in MM.

## Author Contributions

**Reema Patel:** writing – original draft, writing – review and editing, data curation. **Louisa Post‐Zwicker:** writing – original draft, writing – review and editing, data curation. **Varinder Kaur:** conceptualization, writing – review and editing, supervision, data curation.

## Funding

The authors have nothing to report.

## Ethics Statement

All research was conducted in a HIPAA compliant manner and in accordance with the Declaration of Helsinki.

## Consent

The patient provided informed consent for publication of the case report.

## Conflicts of Interest

The authors declare no conflicts of interest.

## Data Availability

The data that support the findings of this study are available on request from the corresponding author. The data are not publicly available due to privacy or ethical restrictions.
